# FBXO45 is a potential therapeutic target for cancer therapy

**DOI:** 10.1038/s41420-020-0291-2

**Published:** 2020-07-03

**Authors:** Min Lin, Zhi-wei Wang, Xueqiong Zhu

**Affiliations:** 1 Departmant of Obstetrics and Gynecology, The Second Affiliated Hospital of Wenzhou Medical University, Wenzhou, Zhejiang 325027 China; 2 Center of Scientific Research, The Second Affiliated Hospital and Yuying Children’s Hospital of Wenzhou Medical University, Wenzhou, Zhejiang 325027 China

**Keywords:** Oncogenes, Targeted therapies

## Abstract

FBXO protein 45 (FBXO45), a substrate-recognition subunit of E3 ligases, has been characterised to have pivotal roles in many human diseases, including nervous system diseases, inflammatory diseases and human malignancies. In this article, we describe the expression of FBXO45 in several types of human tumour specimens and highlight the downstream substrates of FBXO45. Moreover, the biological functions of FBXO45 in the regulation of proliferation, apoptosis, the cell cycle and metastasis are mentioned. Furthermore, we describe that the expression level of FBXO45 is regulated by several upstream factors such as miR-27a, Hey1, m^6^A and the lncRNA RP11. As FBXO45 has a critical role in tumorigenesis and progression, FBXO45 might be a novel therapeutic target for cancer treatment.

## Facts

- FBXO45 exerts its functions by targeting substrates for ubiquitination and degradation.
- FBXO45 has a pivotal role in carcinogenesis and progression.
- Targeting FBXO45 might be a novel strategy for cancer therapy.

## Open questions

- What are the physiological functions of FBXO45 in a variety of human malignancies?
- What are unknown targets of FBXO45 that are critically involved in tumorigenesis?
- How can a novel approach to identify new substrates of FBXO45 be established?

## Introduction

The ubiquitin proteasome system (UPS), which applies posttranslational modifications (PTMs) to proteins, is a vital pathway that drives protein degradation in cells^1^. It is responsible for ~80% of intracellular protein degradation and subsequently modulates a series of biological procedures, such as transcription, mitosis, cell cycle, proliferation, apoptosis, genomic stability and signalling pathways^2,3^. Two well-defined steps are implicated in UPS-mediated protein degradation^4,5^. Primarily, the substrate protein is labelled by ubiquitination (monoubiquitination or polyubiquitination) by three-step enzymatic reactions involving an E1 ubiquitin activating enzyme, an E2 ubiquitin conjugating enzyme, and an E3 ubiquitin ligase. Subsequently, the ubiquitinated substrate is degraded by the 26S ribosomal proteasome complex. Mechanistically, the E3 ubiquitin ligase specifically recognises numerous substrates for ubiquitination and degradation^6^. The Cullin-RING ligase complex family, which contains Skp1-Cullin1-F-box protein (SCF)-type ligases composed of Skp1, Cullin1 (Cul1), Rbx1 and an F-box protein, is one of the large E3 enzyme families^4^. Known to be subunits of the SCF E3 ligase complex, F-box proteins are generally categorised into three subfamilies, including FBXW (F-box with WD 40 amino-acid repeats), FBXL (F-box with leucine-rich amino-acid repeats) and FBXO (F-box only with uncharacterised domains)^7^. F-box proteins have been reported to participate in the development of many diseases, including cancer.

## Role of FBXO45 in human disease

FBXO45, a member of the FBXO protein subfamily, was originally categorised as an oestrogen-induced protein in 2005^8^. Several identical sequences for oestrogen receptor (ER) binding were identified near the transcription start site in both the human and mouse FBXO45 gene. Likewise, FBXO45 mRNA levels were strikingly increased in breast cancer MCF-7 cells after 17β-oestradiol exposure^8^. However, other studies showed that the FBXO45 mRNA level in the liver of mature male zebrafish was not regulated significantly with 17α-ethinyloestradiol (EE2) exposure^9^. The oestrogen/bazedoxifene tissue-selective oestrogen complex (TSEC) construct was designed not only to improve the safety of oestrogen treatment in the endometrium and breast but also to allow the valuable effects of oestrogen to be realised in other oestrogen-targeted tissues, including bone and brain^10^. Mechanistically, the effects of TSEC treatment in the endometrium and breast were proposed to be a result of the repression of ERα-mediated transcription and the promotion of ERα protein ubiquitination and degradation through FBXO45 in uterine tissue and breast cancer cells, but not in bone cells^10^, indicating that FBXO45 has a regulatory role in TSEC-mediated ERα degradation in endometrial and breast cells^10^.

Subsequently, a growing body of data have verified that FBXO45 is closely related to the development of the nervous system^11,12^. One study from the Nakayama group demonstrated that FBXO45 deletion in mice led to death owing to respiratory distress and inappropriate development of the nervous system soon after mice were born^11^. FBXO45 has a crucial role in neural development by establishing the FBXO45-PAM complex^11^. In line with this, another group further uncovered that FBXO45-regulated neurotransmission via degradation of Munc13-1, a synaptic vesicle-priming factor at the synapse, indicating that FBXO45 controls synaptic activity^12^. Notably, in addition to the low expression of spinal FBXO45 that was observed in neuropathic injury, focal loss of spinal FBXO45 also led to increased behaviour allodynia in juvenile animals^12^. Moreover, spinal TNF-α impaired FBXO45-mediated Munc13-1 degradation, resulting in neuropathic allodynia, which could be reversed by an intrathecally administered TNF-α-neutralising antibody^13^. Furthermore, FBXO45 was identified to be critically involved in schizophrenia owing to the R108C mutation of FBXO45, which impairs the FBXO45 function, indicating that FBXO45 might be a useful biomarker for schizophrenia^14^. In addition, Chung et al. found that FBXO45 directly interacted with N-cadherin but suppressed proteolysis of N-cadherin, leading to enhancement of neuronal differentiation^15^. This is different from the universal recognition that F-box proteins have a biological role by targeting their downstream substrate proteins for ubiquitination and degradation. One genome-wide association study was performed to evaluate five coping behaviours: emotional expression, emotional support seeking, positive reappraisal, problem solving and disengagement^16^. Ultimately, FBXO45 was distinctly correlated with emotional expression by regulating synapse maturation^16^. Moreover, FBXO45-targeted NMNAT2 for degradation by forming a complex, PAM/FBXO45/SKP1, and subsequently regulated axon degeneration^17,18^.

In addition, emerging evidence has shown that FBXO45 is germane to other human diseases. The 14 different genetic products originating from 14 single-nucleotide polymorphisms were analysed to find the correlation between FBXO45 and vitiligo in a Chinese Han cohort^19^. Ultimately, it was found that the FBXO45 and NRROS genes were positively correlated with vitiligo^19^. Mass spectrometry was conducted to identify proteins immunoprecipitating with nitric oxide synthase 2 (NOS2) in A549 cells with catalytically inactive NOS2 overexpression, and FBXO45 was newly found to directly interact with NOS2^20^. This may indicate that FBXO45 is associated with inflammatory respiratory disease. These reports indicate the critical role of FBXO45 in noncancerous diseases.

## Role of FBXO45 in cancer

Recently, FBXO45 has also been identified to have a pivotal role in tumorigenesis and progression. In the following paragraphs, we will describe the expression of FBXO45 in human tumour specimens and identify substrates of FBXO45 in cancer and its biological functions in the regulation of proliferation, apoptosis, cell cycle, motility and metastasis.

## FBXO45 is highly overexpressed in human cancer tissues

A wealth of studies have uncovered that FBXO45 could have important roles in tumorigenesis and progression. Data from The Cancer Genome Atlas (TCGA) and GTEx, termed GEPIA, show that FBXO45 is highly expressed in a majority of human cancers (Supplementary Fig. 1). Compared with that in normal lung tissue, FBXO45 is highly expressed in squamous-cell lung carcinoma (SCLC) tissues according to TCGA and Gene Expression Omnibus data^21^. Compared with that in adjacent tissues, an increased level of FBXO45 was confirmed in SCLC tissues by reverse transcription polymerase chain reaction analysis. Furthermore, it was also illustrated that high expression of FBXO45 was correlated with poor survival in patients with SCLC by obtaining data from the Kaplan–Meier plotter website and the TCGA database^21^. Consistent with the results in SCLC, FBXO45 expression was higher in gastric cancer tissues than in normal gastric tissues^22^. However, gastric cancer patients with low FBXO45 expression exhibited poorer outcomes, such as worse survival, than those with high FBXO45 expression^22^. Consistently, data from TCGA show that high expression of FBXO45 is correlated with shortened overall survival in multiple types of human cancers (Supplementary Fig. 2). Moreover, Dahlem et al.^23^ proved that overexpression of insulin-like growth factor 2 (IGF2) mRNA-binding protein IMP2 (IGF2BP2) existed and was associated with poor outcomes in pancreatic cancer patients by investigating publicly available datasets FBXO45 is positively associated with IMP2 expression, indicating that FBXO45 might play a potential oncogenic role in pancreatic cancer progression^23^.

## Identified substrates and functions of FBXO45

Several substrates of FBXO45 have been identified in recent years. For example, FBXO45 targets p73, which belongs to the p53 family, for ubiquitylation and degradation, leading to a reduction in cell death^24^. Consistently, deletion of FBXO45 led to an accumulation of p73 and subsequently triggered cell death in cells^24^. Another study validated that prostate apoptosis response protein 4 (Par-4), an anticancer protein that induces apoptosis, was a downstream substrate of FBXO45 in cancer cells^25^. Specifically, FBXO45 mediated the ubiquitylation and proteolysis of Par-4, leading to a reduction in cell apoptosis. In line with this finding, depletion of FBXO45 led to elevated apoptosis due to upregulation of Par-4. This study indicated that FBXO45 could promote cell survival in human cancer cells^25^. Keeping abreast with this, other studies further demonstrated that the VASA segment is a crucial portion of Par-4 that can bind with FBXO45 and subsequently lead to Par-4 degradation. In contrast, a Par-4 amino-terminal fragment (PAF), generated by therapy-sensitive cancer cells and containing this VASA segment, recovered Par-4-mediated apoptosis by competitively binding FBXO45^26^.

Richter et al.^27^ identified FBXW7 as a new substrate of FBXO45 and dissected the mechanism of cell fate decisions in cancer cells. FBXW7 has been well characterised as a tumour suppressor in carcinogenesis and progression^28^. It has been accepted that FBXW7 exerts its tumour-suppressive function by targeting its substrates for ubiquitination and degradation, including Notch, c-Jun, cyclin E, c-Myc and Mcl-1. FBXW7 is potentially involved in the regulation of multiple cellular processes including cell proliferation, apoptosis, migration, invasion and metastasis. Accumulated evidence has also revealed that FBXW7 governs the epithelial-to-mesenchymal transition (EMT), stem cell differentiation and drug resistance in tumour cells^28^. Numerous upstream regulators of FBXW7 have also been discovered such as p53, Numb, microRNAs and Pin-1^28^. In one study, FBXO45 was validated as an F-box protein targeting another F-box protein, FBXW7, for proteolysis^27^. First, the authors measured the protein levels of FBXW7 in cells during prolonged mitotic arrest and found that FBXW7 expression was reduced in this process. Second, coimmunoprecipitation analysis showed that the N-terminal domain of FBXW7 interacted with FBXO45 and the MYCBP2 complex. Third, the results from immunoblotting and in vivo ubiquitylation demonstrated that FBXO45-MYCBP2 triggered FBXW7 ubiquitination and proteolysis during mitotic arrest. Fourth, using live-cell imaging analysis, the FBXO45-MYCBP2 complex was shown to decrease the cellular sensitivity to spindle poisons, including nocodazole, Taxol and vincristine^27^. Moreover, this complex inhibited mitotic cell deaths and promoted mitotic slippage mainly by targeting FBXW7^27^. Therefore, this study revealed that blockade of FBXO45-mediated FBXW7 degradation might be useful for enhancing mitotic cell death to overcome drug resistance during chemotherapeutic treatment^27^.

**Fig. 1:**
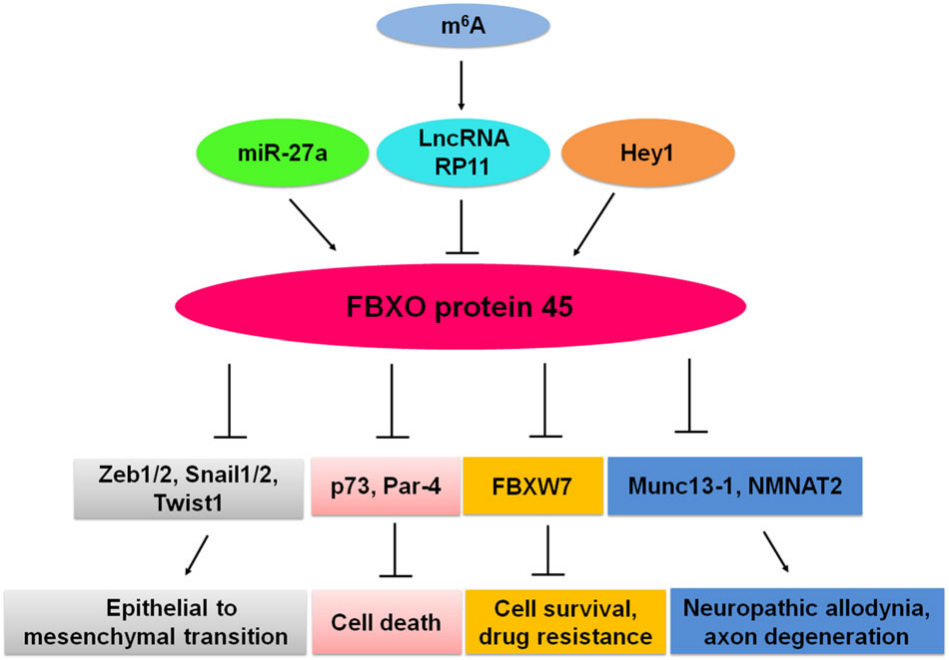
Molecular mechanism of FBXO45 in controlling cellular functions via targeting multiple substrates. FBXO45 targets numerous substrates for ubiquitination and degradation, including FBXW7, p73, Par-4 and ZEB1/2. Hey1, miR-27a and m^6^A regulate the expression of FBXO45. FBXO45 has a critical role in cell death, apoptosis, EMT and drug resistance.

However, other reports indicate that FBXO45 might have an inverse role in specific types of human malignancies. For example, ZEB1, a well-known marker related to EMT, was degraded via the caspase-8-associated protein 2 (CASP8AP2 or FLASH)-dependent SIAH1 E3 ubiquitin ligase and the FBXO45 atypical E3 ligase^29^. Silencing SIAH1 or FBXO45 restored the expression of ZEB1 protein^29^. Likewise, FBXO45 inhibited EMT by targeting EMT-inducing transcription factors, including Zeb1/2, Snai1/2 and Twist1, for ubiquitination and degradation in cancer cells^30^. FBXO45 was related to several biological processes, including cell adhesion, immune response, cell signal transduction and angiogenesis, in GO and KEGG pathways analyses^21^. Interestingly, a study in SCLC further revealed that diminished expression of FBXO45 blocked the colony forming ability of H520 cells, but markedly augmented migration and prompted EMT^21^. Although these studies explored the molecular mechanisms of FBXO45-related tumorigenesis and progression (Fig. 1), the underlying and comprehensive mechanisms have not been fully elucidated, which is required for in-depth exploration.

## Upstream regulators of FBXO45

A study showed that miR-27a could inhibit the expression of FBXO45, leading to accumulation of the downstream substrates of FBXO45 and modulation of cancer initiation and progression, indicating that FBXO45 is a direct target of miR-27a^30^. One group revealed that Hey1 could promote FBXO45 translocation from the cytoplasm into the nucleus, indicating that Hey1-mediated translocation of FBXO45 could be a useful way to degrade the nuclear substrates of FBXO45^31^. One elegant study revealed that lncRNA RP11-138 J23.1 (RP11) was overexpressed in colorectal cancer compared with matched normal colorectal tissues by microarray analysis^32^. Furthermore, they found that the expression of RP11 was associated with the progression of colorectal cancer stage. Further investigations showed that m^6^A upregulated the lncRNA RP11 via promotion of its nuclear accumulation and subsequently enhanced the mRNA degradation of FBXO45, eventually preventing the ZEB1 degradation, inducing EMT and increasing migration and invasion in colorectal cancer^32^. An amino-terminal of PAF could competitively bind to FBXO45, resulting in an increase in cell apoptosis owing to Par-4 accumulation. This PAF induced apoptosis and suppressed tumour growth in drug-resistant cells, suggesting that it could overcome drug resistance in cancer therapy^26^. Therefore, modulation of miR-27a, Hey1, m^6^A and RP11 could affect the FBXO45 expression level as a potential approach to treat cancer patients with high FBXO45 expression (Fig. 1).

## Conclusion and perspective

In summary, FBXO45 is critically involved in carcinogenesis and cancer progression by targeting its downstream substrates for ubiquitination and degradation (Table 1). Targeting FBXO45 with inhibitors might be a novel strategy for achieving a therapy benefit in cancer patients. One alternative approach is to target the upstream regulators of FBXO45, leading to regulation of the FBXO45 expression level. It is worth noting that several critical questions need to be addressed to fully understand the role of FBXO45 in carcinogenesis and cancer progression. For instance, what are the biological functions of FBXO45 in other types of human malignancies that have not been reported? To answer this, it is better to use conditional transgenic knock-in or knockout mouse models to determine the role of FBXO45 in specific tissues. In addition to the several substrates of FBXO45 that are mentioned above, what are other targets of FBXO45 in human cancer cells? How can a novel approach for identifying new substrates of FBXO45 be established? It is also necessary to explore the upstream factors controlling FBXO45 expression, which will help us to fully dissect the crosstalk between FBXO45 and other signalling pathways. Because the implications for the therapeutic targeting of FBXO45 are not obvious, in-depth exploration is required to uncover the functions of FBXO45 and the underlying molecular mechanisms to develop effective inhibitors of FBXO45 for the treatment of human diseases.

**Table 1:** FBXO45 targets substrates for degradation in human diseases.

Substrates	Cell lines	Functions	Reference
ERα	HeLa, MCF-7	Involves in tissue-selective oestrogen complex-mediated endometriosis therapy	^10^
Munc13-1	293T, hippocampal neuron cells, COS.	Controls synaptic activity, neuropathic allodynia	^12,13^
NMNAT2	293T	Regulates axon degeneration	^17,18^
p73	BT-20, 293T, HeLa	Reduction of cell death	^24^
Par-4	HeLa, 293T	Reduction of cell apoptosis, promotes cell survival	^25^
FBXW7	U2OS, HeLa	Governs cell fate decision, chemotherapy resistance	^27^
ZEB1/2, Snail1/2, Twist1	293T, HeLa, U2OS, MCF-7, MDA-MB-231, PANC-1	Regulates EMT	^29,30^

## Supplementary information

Supplementary figure 1

Supplementary figure 2

Supplementary Figure legend
