# Supplementary figures and images for: FBXO45 is a potential therapeutic target for cancer therapy
Source: Cell Death Discov. 2020 Jul 3;6:55. doi: 10.1038/s41420-020-0291-2 (PMC7335190; doi:10.1038/s41420-020-0291-2)

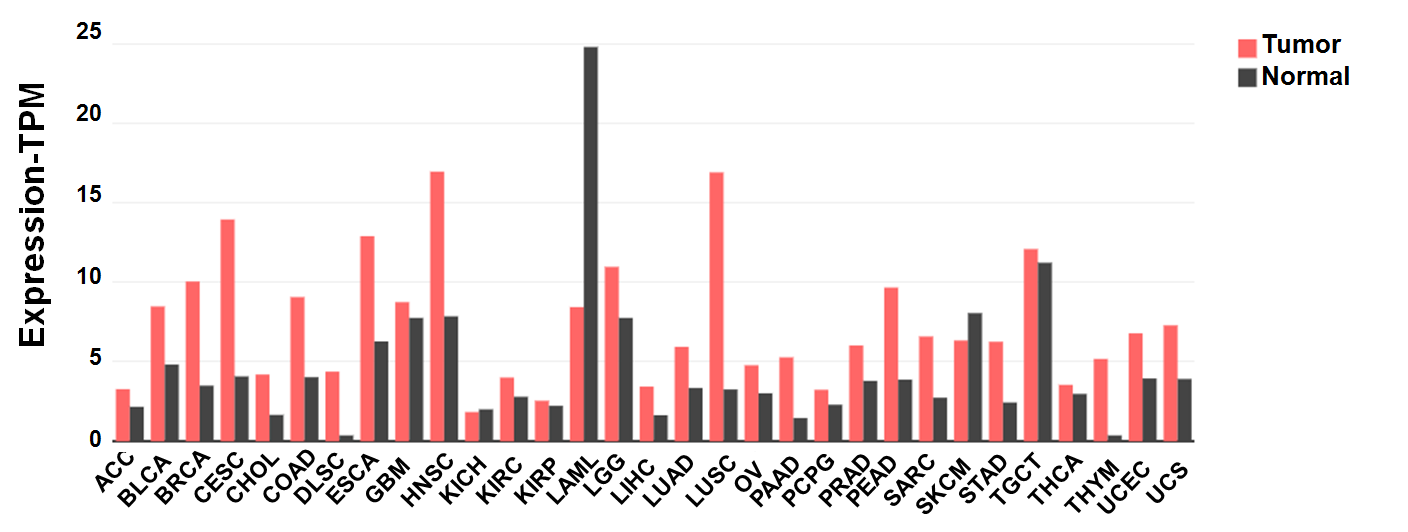

Supplement: Supplementary file 1 — Supplementary figure 1 [file 41420_2020_291_MOESM1_ESM.tif]

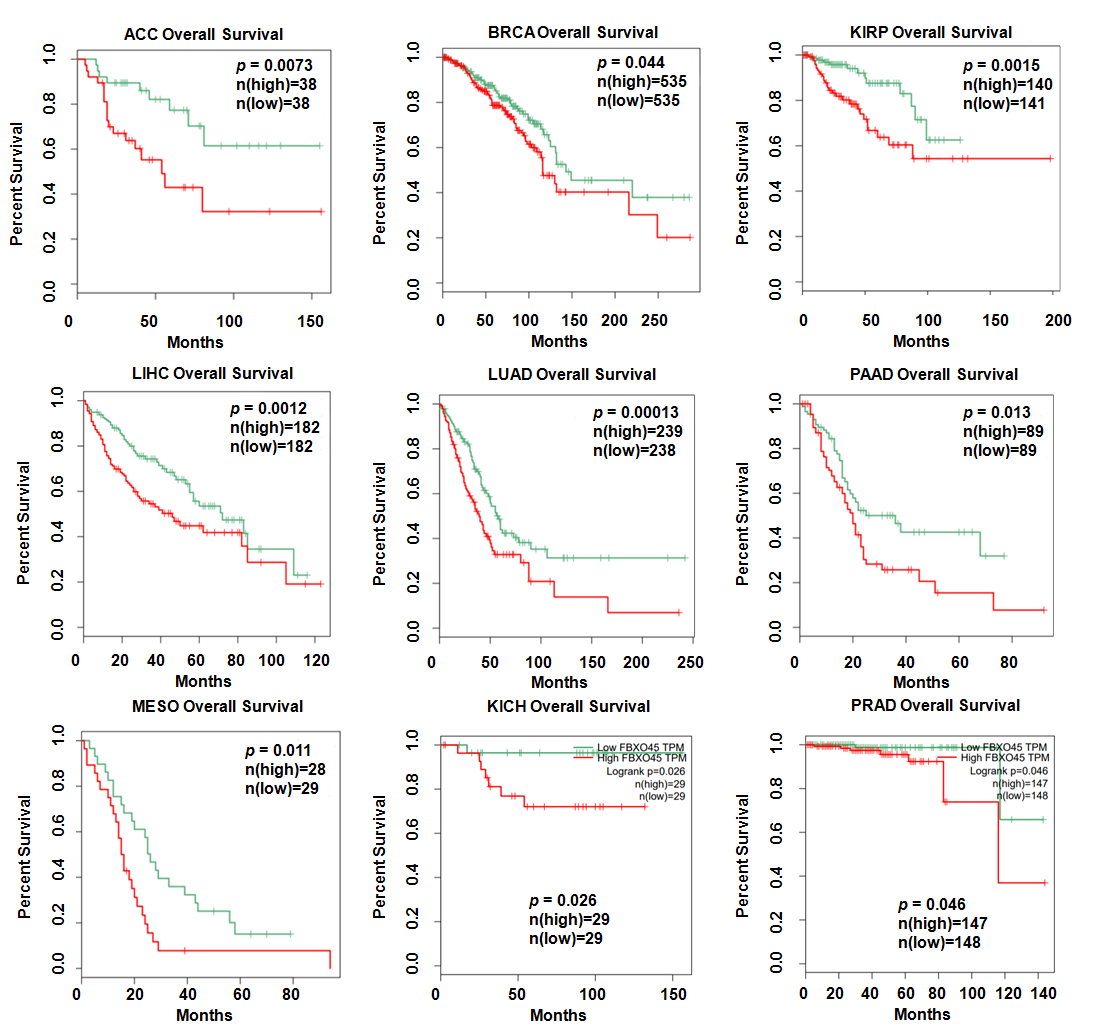

Supplement: Supplementary file 2 — Supplementary figure 2 [file 41420_2020_291_MOESM2_ESM.tif]
