# Supplementary material for: FBXO45 is a potential therapeutic target for cancer therapy
Source: Cell Death Discov. 2020 Jul 3;6:55. doi: 10.1038/s41420-020-0291-2 (PMC7335190; doi:10.1038/s41420-020-0291-2)
Supplement: Supplementary file 3 — Supplementary Figure legend [file 41420_2020_291_MOESM3_ESM.docx]

**Supplementary Figure Legends:**

**Supplementary Figure 1: The FBXO45 gene expression in human tumor samples and normal tissues.** The height of bar represents the median expression of certain tumor type or normal tissue. The data are from GEPIA (TCGA+GTEx ) cite. TPM: Transcripts per million. ACC: Adrenocortical carcinoma, BLCA: Bladder Urothelial Carcinoma, BRCA: Breast invasive carcinoma, CESC: Cervical squamous cell carcinoma and endocervical adenocarcinoma, CHOL: Cholangio carcinoma, COAD: Colon adenocarcinoma, DLBC: Lymphoid Neoplasm Diffuse Large B-cell Lymphoma, ESCAE: esophageal carcinoma, GBM: Glioblastoma multiforme, HNSC: Head and Neck squamous cell carcinoma, KICH: Kidney Chromophobe, KIRC: Kidney renal clear cell carcinoma, KIRP: Kidney renal papillary cell carcinoma, LAML: Acute Myeloid Leukemia, LGG: Brain Lower Grade Glioma, LIHC: Liver hepatocellular carcinoma, LUAD: Lung adenocarcinoma, LUSC: Lung squamous cell carcinoma, MESO: Mesothelioma, OV: Ovarian serous cystadenocarcinoma, PAAD: Pancreatic adenocarcinoma, PCPG: Pheochromocytoma and Paraganglioma, PRAD: Prostate adenocarcinoma, READ: Rectum adenocarcinoma, SARC: Sarcoma, SKCM: Skin Cutaneous Melanoma, STAD: Stomach adenocarcinoma, TGCT: Testicular Germ Cell Tumors, THCA: Thyroid carcinoma, THYM: Thymoma, UCEC: Uterine Corpus Endometrial Carcinoma, UCS: Uterine Carcinosarcoma.

**Supplementary Figure 2: FBXO45 expression is associated with poor survival in a variety of human cancers.** Green line: Low FBXO45; Red line: High FBXO45. ACC: Adrenocortical carcinoma, BRCA: Breast invasive carcinoma, KICH: Kidney Chromophobe, KIRP: Kidney renal papillary cell carcinoma, LIHC: Liver hepatocellular carcinoma, LUAD: Lung adenocarcinoma, MESO: Mesothelioma, PAAD: Pancreatic adenocarcinoma, PRAD: Prostate adenocarcinoma.
